# Understanding the burden and impact of methicillin-resistant *Staphylococcus aureus*: exploring therapeutic options and hospital prevention strategies to improve patient outcome at the University Teaching Hospital of Kigali, Rwanda

**DOI:** 10.3389/fpubh.2025.1718336

**Published:** 2026-01-14

**Authors:** Samuel Rutare, Florence Masaisa, Théoneste Nkubana, Angelique Dusabe, Jean Bosco Munyemana

**Affiliations:** 1 Department of Pediatrics, University Teaching Hospital of Kigali, Kigali, Rwanda; 2 Department of Internal Medicine, University Teaching Hospital of Kigali, Kigali, Rwanda; 3 Department of Internal Medicine, School of Medicine and Pharmacy, College of Medicine and Health Sciences, University of Rwanda, Kigali, Rwanda; 4 Department of Pathology, University Teaching Hospital of Kigali, Kigali, Rwanda; 5 Department of Microbiology and Parasitology, School of Medicine and Pharmacy, College of Medicine and Health Sciences, University of Rwanda, Kigali, Rwanda

**Keywords:** length of hospital stay, MDR, methicillin-resistant *Staphylococcus aureus*, MRSA, patient outcomes

## Abstract

**Background:**

Methicillin-resistant *Staphylococcus aureus* (MRSA) remains a significant global public health challenge, particularly as a leading cause of nosocomial infections in intensive care units (ICUs). While MRSA prevalence is well documented in many settings, its burden at the University Teaching Hospital of Kigali (CHUK), Rwanda, remains unclear.

**Aim:**

To evaluate the burden and impact of MRSA, exploring therapeutic options and hospital prevention strategies to improve patient outcomes.

**Method:**

A cross-sectional descriptive study was conducted at CHUK from 1st July 2024 to 30th June 2025 at the University Teaching Hospital of Kigali, in Rwanda. All patients with clinical samples positive for *Staphylococcus aureus* (*S. aureus*) were included. Antimicrobial susceptibility patterns of isolates were analyzed, and patient clinical records were reviewed.

**Results:**

During the study period, 272 *S. aureus* isolates were analyzed, of which 117 (43%) were MRSA. The isolates were highly resistant against commonly used antibiotics, including ampicillin (92.7%), erythromycin (49.3%), ciprofloxacin (42.3%), and tetracycline (48.5%). However, they demonstrated high sensitivity to vancomycin, linezolid, teicoplanin, amikacin, daptomycin, and rifampicin.

**Conclusion:**

*Staphylococcus aureus* infections are common at CHUK, with a high prevalence of MRSA (43%). Effective therapeutic options remain available, but the high resistance to commonly used antibiotics underscores the need for strengthened infection prevention measures and antimicrobial stewardship to improve patient outcomes, especially in critical care services.

## Background

*Staphylococcus aureus (S. aureus)* is a significant cause of community- and hospital-acquired infections. Methicillin-resistant *S. aureus* (MRSA) is defined as *S. aureus* that is resistant to beta-lactam antibiotics, including methicillin and other more commonly used antibiotics, such as oxacillin, penicillin, and amoxicillin (1, 2). MRSA was first described in 1961 (3), and since then, it has become a significant human pathogen. The first reports of MRSA infection were from Boston City Hospital in 1968 (4). MRSA colonization or infection varies between regions, types of health care facilities, and specific populations being studied. MRSA infections are usually hospital-acquired and are increasingly reported from different areas of the world (5, 6).

MRSA can be resistant to many different classes of antibiotics, other than just beta-lactams, and therefore represents a growing concern (7, 8). *S. aureus* isolates have become resistant to even Vancomycin, the last resort drug for MRSA infection globally (9, 10). Multidrug-Resistant *S. aureus* is defined as an isolate that has acquired non-susceptibility to at least one molecule in three or more antibiotic categories (11).

The resistance of *S. aureus* against methicillin is caused by expression of Penicillin Binding Protein 2A (PBP2A) encoded by the *mecA* gene (12). This protein, which has low affinity for beta-lactam antibiotics such as amoxicillin, methicillin, and oxacillin, renders these antibiotics ineffective in treating infections caused by *S. aureus*. The origin of the *mecA* gene is not known, but evidence supports horizontal transfer between different *staphylococcal species* and other Gram-positive genera. The *mecA* gene in *S. aureus* is located on the genetic element *staphylococcal* cassette chromosome (SCC) (12, 13).

Methicillin-resistant *Staphylococcus aureus* infections are mainly nosocomial and are a result of healthcare-related procedures. Currently, MRSA is the most commonly identified antibiotic-resistant bacterium in many different countries (5). Its prevalence has been increasing, as shown by the data from continuing surveillance initiatives such as the National Nosocomial Infection Surveillance System and European Antimicrobial Resistance Surveillance System. A study conducted amongst the Nordic countries has shown that the prevalence of MRSA has been low in the past, but now has begun to rise. In 2016, Denmark had the highest incidence of 62 new cases per 100,000 people, and MRSA-spa typing showed high genetic diversification (6, 14).

Although MRSA prevalence data exist for many countries, heterogeneity in study designs, institutional inclusion criteria, antibiotic susceptibility testing, and specimen selection complicates international comparisons. Data from Africa remains limited, particularly regarding MRSA antibiotic sensitivity patterns (5). MRSA surveillance plays a crucial role in understanding epidemic burden and guiding infection control and antimicrobial policies (5). Hospital-acquired MRSA infections increase morbidity, mortality risk, healthcare costs, and productivity loss (5). Antimicrobial resistance poses a global health threat, with an estimated five million deaths attributable to it in 2019, over 100,000 of which were caused by MRSA (15).

There is a paucity of data on MRSA prevalence and its antimicrobial susceptibility pattern in Rwanda. Thus, this study aimed to evaluate the burden and impact of MRSA, exploring therapeutic options and recommendations for hospital prevention strategies to improve patient outcomes.

## Method

### Study design

This was a one-year prospective descriptive and analytical study conducted from 1st July 2024 to 30th June 2025.

### Study site

The study was carried out at the University Teaching Hospital of Kigali, also known as the Center Hospitalier Universitaire de Kigali (CHUK). CHUK is the largest tertiary national referral and teaching hospital located in Nyarugenge District, Kigali, Rwanda. Established in 1918, CHUK has evolved into the premier referral healthcare institution with a bed capacity of approximately 500 beds. It receives around 16,000 to 18,000 inpatient admissions annually and maintains a bed occupancy rate between 80 and 90%. The average patient length of stay is about 7 days. CHUK’s core mission is to provide high-quality specialized healthcare services, promote medical education, advance clinical research, and support health system strengthening across Rwanda. Through partnerships and outreach, CHUK aims to decentralize advanced healthcare services to provincial and district hospitals, improving accessibility and equity for the entire population.

### Study population and sample size

The study included all consecutive patients who visited CHUK with signs of sepsis, infected wounds (surgical or non-surgical), abscesses, osteomyelitis, septic arthritis, or pleural and pericardial effusions. The sample size was calculated as per Fisher’s formula for calculating sample size using precision around a proportion, which provided the minimum sample required, where the total sample size calculated was 287. However, during a one-year study period, we obtained 272 *S. aureus* cases, which were included in the data analysis.

### Inclusion criteria

All patients visited CHUK during the study period with clinical signs of sepsis, infected wounds, abscesses, osteomyelitis, septic arthritis, or pleural/pericardial effusions from whom clinical samples for culture were obtained were eligible. Moreover, outpatients were also included if *S. aureus* was isolated. The outpatients were defined as those receiving care without hospital admission, typically assessed and treated on the day of presentation at CHUK and returned home the same day. Written informed consent was obtained from the participants or the parent/guardian of the children before enrollment.

### Exclusion criteria

Patients with signs of infection for whom clinical samples could not be obtained or who declined consent were excluded from the study. Moreover, the patients whom other than *S. aureus* were isolated from their samples, were not considered for statistical analysis.

### Recruitment procedures, data collection, and sampling

Data collection was conducted over the 12-month study period. The principal investigator (PI) made daily ward visits to identify and recruit eligible participants. After explaining the study objectives and procedures to parents or guardians, written informed consent was obtained. Clinical samples, including blood, pus, tracheal aspirates, cerebrospinal fluid (CSF), pleural or pericardial fluids, were collected according to clinical indications.

For invasive samples such as deep abscesses, osteomyelitis, septic arthritis, pleural, or pericardial effusions, collaboration with surgical and orthopedic teams ensured appropriate sample collection. Tracheal aspirates were obtained from intubated patients.

Samples were transported promptly to the microbiology laboratory for culture and antimicrobial susceptibility testing. Sociodemographic and clinical data were collected using structured questionnaires through reviews of patient files, outpatient clinic records, and laboratory reports. To ensure confidentiality, data were anonymized by assigning study identification numbers. A separate secure log linking patient identifiers to study numbers was maintained solely by the PI for follow-up purposes.

The PI was supported by trained research assistants in data collection. Nurses on duty were formally requested to notify the PI of new admissions and the availability of parents/guardians for consent.

### Culture and antimicrobial susceptibility testing

Specimens were cultured on mannitol salt agar (MSA) and sheep blood agar (SBA) and incubated aerobically at 37 °C for 18 to 24 h. Colonies demonstrating yellow coloration on mannitol agar and *β*-hemolysis on SBA were subjected to catalase tests. Catalase-positive isolates were Gram-stained and further identified using the coagulase test. Confirmed *S. aureus* isolates underwent antimicrobial susceptibility testing using the disk diffusion method.

The following antibiotic discs were tested: oxacillin (OX): 1 μg, vancomycin (VA): 30 μg, gentamicin (CN):10 μg, amoxicillin-clavulanic acid (AMC): 30 μg, chloramphenicol (C): 30 μg, erythromycin (E): 15 μg, tetracycline (TE): 30 μg, cefotaxime (CTX): 30 μg, ampicillin (AMP):10 μg, amikacin (AK): 30 μg, clindamycin (DA): 2 μg, meropenem (MEM): 10 μg, linezolid (LZD): 30 μg, and sulfamethoxazole-trimethoprim (SXT-TMP): 25 μg, amikacin (AK): 30 μg, doxycycline: 30 μg, rifampin (RIF): 5 μg, nitrofurantoin (F/NIT): 300 μg (BD, 7 Loveton Circle, Sparks, MD 21152, USA).

The susceptibility testing results were interpreted as sensitive, intermediate, and resistant by using the Clinical Laboratory Standards Institute (CLSI) 2024 breakpoint (16). Furthermore, internal quality control was performed by using American Type Culture Collection (ATCC) 25,923 as a positive control (*Staphylococcus aureus*) and ATCC 25922 as a negative control (*Escherichia coli*).

### Data variables

Collected data included demographic characteristics, clinical presentation at admission, admission diagnosis, risk factors, isolated pathogens including MRSA, and antimicrobial susceptibility profiles. Antibiotic susceptibility was classified as sensitive, intermediate, or resistant according to standardized diffusion zone diameters. All data were recorded using structured worksheets and subsequently entered into a computer database for analysis.

### Data analysis

Data was recorded in an Excel sheet and exported to IBM SPSS version 28.0 (BM Corp., Armonk, NY, USA) for statistical analysis. Descriptive statistics, including frequencies, proportions, and means, were calculated. Group comparisons were performed using Student’s *t*-test or non-parametric equivalents where data normality was not met. Statistical significance was set at *p* < 0.05. Results were presented in tables, bar charts, and line graphs as appropriate.

### Ethical considerations

Ethical approval for the study was obtained from the Kigali University Teaching Hospital Ethics Board. The study objectives and procedures were fully explained to parents or guardians, and written informed consent was secured before participant enrollment. Confidentiality of participant information was strictly maintained, with data securely stored and accessible only to the investigator. Parents were informed that the study’s purpose was to generate knowledge to improve clinical care for patients admitted to the CHUK pediatric wards.

## Results

### Demographic and clinical characteristics

This study has included 272 patients infected with *S. aureus*, with a slight male predominance (53.7% male vs. 46.3% female). The population was relatively young, with most patients aged 25–44 years (34.2%), followed by 5–14 years (16.2%) and under 5 years (15.4%), while only 8.5% were 65 years or older. The mean and median ages were 25 and 20 years, respectively. The study participants were from different Departments, including Pediatrics (34.2%), Surgery (20.2%), Internal Medicine (12.5%), ICU (11.4%), and A&E (11.8%), with smaller proportions from G&O, Neonatology, and OPD.

*Staphylococcus aureus* isolates were mostly isolated from blood cultures (57.0%), followed by pus cultures (34.2%). Overall, MRSA was identified in 117 of 272 *S. aureus* cases (43%), while 155 (57%) were methicillin-susceptible *S. aureus* (MSSA). Among 246 cases of inducible clindamycin test (*D* test) performed, one-third (33%) were positive. Moreover, among 270 study participants whose clinical outcome was investigated, 228 (84.4%) have recovered, while 42 (15.6%) died (Table 1).

**Table 1:** Demographic and clinical characteristics.

Variable	Category	Frequency	Percentage
Sex (*n* = 272)	Male	146	53.7
	Female	126	46.3
Age (Years)	Mean age in years	25	–
	Median in years	20	–
	Standard Deviation	23	–
Age Group (*n* = 272)	<5 years	42	15.4
	5–14 years	44	16.2
	15–24 years	37	13.6
	25–44 years	93	34.2
	45–64 years	33	12.1
	65 + years	23	8.5
Department (*n* = 272)	Pediatrics	93	34.2
	ICU	31	11.4
	Surgery	55	20.2
	A&E	32	11.8
	IM	34	12.5
	G&O	11	4.0
	Neonatology	5	1.8
	OPD	11	4.0
Sample types (*n* = 272)	Blood	155	57
	Pus	93	34.2
	Other	24	8.8
MRSA (*n* = 272)	Positive	117	43
	Negative	155	57
D T test (*n* = 246)	Positive	81	33
	Negative	165	67
Outcome (*n* = 270)	Cured	228	84.4
	Died	42	15.6

Data are presented as frequency and percentages unless otherwise indicated.

### Overall antimicrobial resistance profile in *S. aureus* isolates

**Figure 1:**
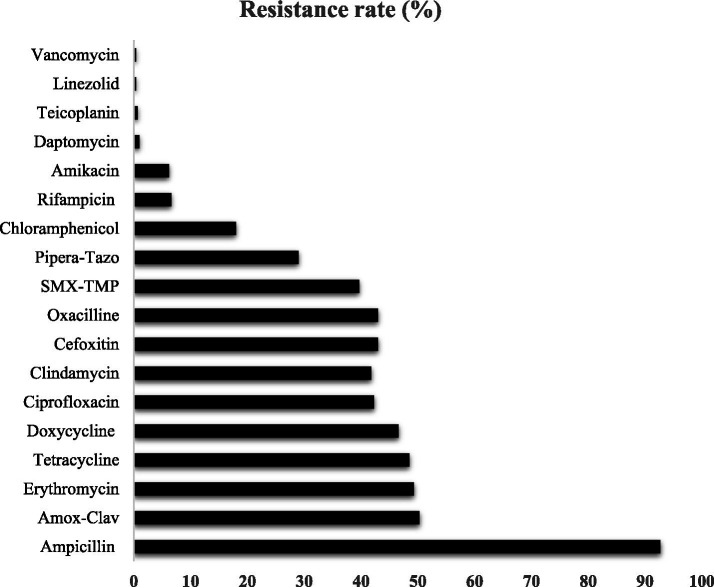
Antimicrobial resistance pattern in all *Staphylococcus aureus* isolates (*N* = 272). Amox-Clav: Amoxicillin-Clavulanic acid; Pipera-Tazo: Piperacillin/Tazobactam; SMX-TMP: Sulfamethoxazole/Trimethoprim.

Among all *Staphylococcus aureus* isolates, a very high resistance was observed to ampicillin (92.7%), with moderate resistance to amoxicillin – clavuranic acid (50.3%), erythromycin (49.3%), tetracycline (48.5%), doxycycline (46.6%), ciprofloxacin (42.3%), clindamycin (41.8%), cefoxitin (43%), oxacillin (43%), and trimethoprim–sulfamethoxazole (39.7%). Lower resistance rates were noted for piperacillin–tazobactam (29%) and chloramphenicol (18%), while rifampicin (6.6%) and amikacin (6.2%) retained good activity. Resistance to last-line agents remained rare, with only 1% for daptomycin and below 1% for teicoplanin (0.7%), linezolid (0.4%), and vancomycin (0.4%) (Figure 1).

### The resistance rate among MRSA phenotypes

**Figure 2:**
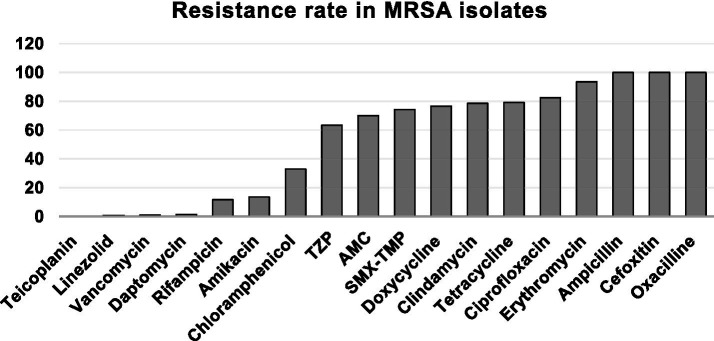
Antimicrobial resistance pattern in MRSA-positive isolates: AMC: amoxicillin-clavulanic acid, TZP: piperacillin/tazobactam; SMX/TMP: sulfamethoxazole/trimethoprim.

Among MRSA isolates, resistance was extremely high to several first-line agents, with 100% of strains resistant to ampicillin, cefoxitin, and oxacillin, and very high resistance observed to erythromycin (93.4%), tetracycline (79.2%), doxycycline (76.6%), ciprofloxacin (82.5%), clindamycin (78.5%), bactrim (74.2%), and piperacillin–tazobactam (63.4%). Moderate resistance was seen to chloramphenicol (33%), while rifampicin (11.6%) and amikacin (13.5%) retained good activity. Resistance to last-line agents remained rare, with only 1.3% of isolates resistant to daptomycin, 0.8% to linezolid, 0.9% to vancomycin, and no resistance detected to teicoplanin (Figure 2).

### MRSA infection risk across clinical departments

Across departments, non-duplicate MRSA were 112 (42%) of all *S. aureus* isolates, with notable variation in distribution by clinical area. Pediatrics contributed the largest share of all *S. aureus* isolates (34.5%) but had a relatively low and non-significant odds of MRSA compared with the reference (OR 0.76, 95% CI 0.46–1.27, *p* = 0.37). ICU showed both a high proportion of MRSA (20 of 30 isolates) and a significantly increased odds of MRSA (OR 3.17, 95% CI 1.43–7.03, *p* = 0.004). In contrast, Surgery had a significantly lower odds of MRSA (OR 0.51, 95% CI 0.27–0.94, *p* = 0.037), while A&E, Internal Medicine, G&O, Neonatology, and OPD showed no statistically significant associations with MRSA occurrence, despite some departments having relatively small sample sizes that may limit precision (Table 2).

**Table 2:** MRSA infection risk across clinical departments at CHUK.

Department	All *S. aureus*	MRSA	OR	95%CI	*P*-value
	Frequency (%)	Frequency (%)			
Pediatrics	92(34.5)	35(13.1)	0.76	0.46–1.27	0.37
ICU	**30(11.2)**	**20(7.4)**	**3.17**	**1.43–7.03**	**0.004**
Surgery	59(21.7)	18(6.6)	0.51	0.27–0.94	0.037
A&E	32(11.8)	17(6.3)	1.59	0.76–3.33	0.26
IM	27 (10.1)	13(5.0)	1.60	0.75–3.42	0.25
G&O	11(4.0)	3(1.1)	0.48	0.13–1.86	0.36
Neonatology	5(1.8)	3(1.1)	2.01	0.33–12.2	0.65
OPD	11(4.0)	3(1.1)	0.48	0.13–1.86	0.36
Total	267(100)	112(42)			

ICU: intensive care unit; A&E: Accident and emergency; IM: Internal medicine, OR. Odd ratio; CI: Confidence Interval. *p* value less than 0.05 was considered statistically significant.

### Infection and clinical outcomes

Among 117 MRSA-positive isolates, 112 cases were considered for clinical outcome analysis after removing duplicates (more than one isolate from the same individual but isolated in different sample types). MRSA positivity was significantly associated with mortality (*p* = 0.03; adjusted *p* = 0.03). Adjustment for age, sex, length of hospital stay, and *D*-test result did not change this effect, and none of these covariates showed a significant independent association with outcome. The *D*-test result alone was not associated with cure or death (*p* = 0.20; adjusted *p* = 0.20). Similarly, LHS categories showed no statistically significant relationship with outcome (*p* = 0.4; adjusted *p* = 0.3). In contrast, the department of admission was strongly associated with outcome (*p* = 0.002; adjusted *p* = 0.002), with a higher proportion of deaths among ICU patients compared with departments such as Surgery, Neonatology, and OPD, where very few or no deaths were observed (Table 3).

**Table 3:** Association of infection and clinical outcomes.

Variables		Outcome		*p*-value	Ad. Pvalue
Indicator	Results	Cured	Died		
MRSA	Negative	137	18	0.03	0.03
	Positive	90	22		
D test	Negative	141	24	0.2	0.2
	Positive	66	15		
LHS	1–2 days	28	4	0.4	0.3
	3–14 days	70	19		
	15–30 days	48	6		
	>30 days	68	11		
Department	Pediatrics	77	15	0.002	0.002
	ICU	20	10		
	Surgery	54	1		
	A&E	24	8		
	IM	26	5		
	G&O	10	1		
	Neonatology	5	0		
	OPD	11	0		

ICU: intensive care unit; A&E: Accident and emergency; IM: Internal medicine, G&O: Gynecology and Obstetrics; OPD: Outpatient Department; OR. Odd ratio; Ad: Adjusted.

## Discussion

This study reveals a substantial burden of MRSA infections among a predominantly young patient population at a tertiary hospital, with critical implications for clinical management and infection control. MRSA prevalence among *S. aureus* isolates reached 43%, aligning closely with rates reported in Tunisia (41%), Botswana (44%), and the Ivory Coast (39%) (17). Similarly, Wangai et al. documented a 53.4% prevalence at Kenyatta National Hospital in Kenya (18).

In contrast, MRSA prevalence in our study exceeded rates from Masaisa et al. (31.2%) and Gitau et al. (27.8%) (19, 20). These differences likely stem from our broader inclusion of patients across internal medicine, surgery, pediatrics, obstetrics and gynecology, and especially the ICU, a high-risk setting for nosocomial infections over a full year. Masaisa et al. used a shorter collection period, while Gitau et al. spanned 3 years across general wards and the ICU with yearly fluctuations. The escalating MRSA burden may further reflect rising antibiotic misuse and suboptimal infection control practices (21).

The demographic findings reveal that most infections occurred in younger age groups, particularly among patients aged 25–44 years and children under 15 years. This is in line with previous findings in Rwanda (19). The distribution of infections among younger patients likely reflects the hospital’s patient demographics as previously reported and Rwanda’s infection epidemiology, in which infectious diseases disproportionately affect younger populations that predominate the national population, according to the Rwanda National Health Survey of 2019 (22, 23).

Most isolates were identified from blood cultures, indicating a predominance of invasive bloodstream infections in this cohort. Bloodstream infections caused by MRSA are associated with high morbidity and mortality, consistent with the elevated death rates observed among MRSA-infected patients. In multivariable analysis, MRSA infection remained independently associated with mortality after adjusting for confounders, including age, sex, length of hospital stay, and inducible clindamycin resistance. The role of MRSA in bloodstream infections has been documented previously in Rwanda (19), Botswana (24), Ethiopia (25, 26), and elsewhere (27). These findings underscore MRSA as a critical marker of sepsis with poor prognosis, emphasizing the urgent need for enhanced infection prevention measures and targeted antimicrobial stewardship.

The resistance profile in this cohort is alarming. High resistance to ampicillin and 100% resistance to beta-lactams (cefoxitin, oxacillin) among MRSA isolates confirms expected phenotypic patterns, which also had an elevated resistance rate to erythromycin, tetracyclines, and ciprofloxacin, limiting empirical therapy common in Rwanda. However, MRSA showed high susceptibility to rifampicin, amikacin, and last-line agents (daptomycin, vancomycin, linezolid, teicoplanin), preserving therapeutic options. These findings align with prior studies conducted in Rwanda (12, 19, 28–30). Although resistance to these last-line agents was very low, even rare resistance could have severe clinical consequences, emphasizing the importance of antimicrobial stewardship and ongoing resistance surveillance to preserve the future use.

Methicillin-resistant *Staphylococcus aureus* distribution varied across clinical departments at CHUK. The ICU showed significantly higher odds of MRSA, reflecting risks in critically ill patients from invasive devices, prolonged stays, and broad-spectrum antibiotics. Surgery had lower odds, possibly due to differing patient profiles, infection control, or antibiotic prophylaxis before surgical intervention. Pediatrics and Internal Medicine lacked significant associations, likely due to sample size or exposure heterogeneity. These patterns align with previous studies confirming ICU status as a key risk factor (31–34).

In the current study, inducible clindamycin resistance occurred in about one-third of tested isolates, which is consistent with other studies (35–37). Since clindamycin is often used as an alternative agent, particularly in outpatient or less severe infections, the occurrence of inducible clindamycin resistance may lead to treatment failures if not properly detected and reported. Yet, in this study, it was not significantly associated with mortality or cure, suggesting that other factors, like infection severity or site, may have stronger impacts on outcomes.

Clinical outcomes were favorable in 84.4% of participants, with 15.6% mortality strongly linked to MRSA infection and ICU admission. A meta-analysis confirmed ICU admission as a significant mortality risk factor in MRSA bloodstream infections (OR ≈ 3.08; 95% CI 1.49–6.36) (38). Consistent with our findings, a Colombian cohort study (2007–2023) identified MRSA as independently associated with increased ICU admission (39). These results underscore the need for aggressive management of MRSA infections in critical care, where resource constraints and care complexity likely worsen prognosis.

This study has some limitations. Its single-center design and lack of molecular typing data limit our ability to elucidate transmission dynamics and clonal relationships among MRSA isolates across clinical settings. Additionally, although exploring hospital prevention strategies was one of the study’s aims, we could not fully assess their impact. Available guidelines exist but are not implemented uniformly across wards, which may partly explain differences in MRSA prevalence beyond ward-specific factors. Future research should evaluate detailed preventive strategies, such as the effects of specific treatment regimens, antibiotic stewardship interventions, and adherence to infection control protocols.

## Conclusion

In summary, MRS*A* prevalence was 43%, mainly isolated from blood and pus specimens. MRSA isolates demonstrated the highest susceptibility to vancomycin, linezolid, teicoplanin, daptomycin, amikacin, and rifampicin, while showing high resistance to commonly used antibiotics such as ampicillin, amoxicillin-clavulanic acid, and ciprofloxacin. Further studies are warranted to distinguish whether these highly resistant MRSA strains are predominantly hospital-acquired or community-acquired infections.

## Data Availability

The original contributions presented in the study are included in the article/supplementary material, further inquiries can be directed to the corresponding authors.
